# Revealing the surface structural cause of scratch formation on soda-lime-silica glass

**DOI:** 10.1038/s41598-022-06649-y

**Published:** 2022-02-17

**Authors:** Barsheek Roy, Andreas Rosin, Thorsten Gerdes, Stefan Schafföner

**Affiliations:** Keylab Glass Technology, Chair of Ceramic Materials Engineering, University of Bayreuth, Prof. Rüdiger-Bormann-Str. 1, 95447 Bayreuth, Germany

**Keywords:** Nanoscale materials, Structural materials, Techniques and instrumentation

## Abstract

Scratch formation on glass surfaces is a ubiquitous phenomenon induced by plastic deformation, often accompanied by radial, lateral or median cracks with consequent chipping and brittle fracture caused during and after the event of dynamic abrasion instigated by shear stress by a harder material. This paper addresses the fundamental aspect of scratch formation on soda-lime-silica (SLS) glass surfaces. A constructive combination of surface-sensitive characterization tools, including field emission scanning electron microscopy (FESEM), laser scanning microscopy (LSM), X-ray photoelectron spectroscopy (XPS), Raman spectroscopy and instrumented indentation technique (IIT), helped to investigate the structural cause of generation of visible scratches on SLS glass surfaces. The experimental results indicate that a silicate network possessing a mechanically weakening structural characteristic in terms of network connectivity confined to the region between 5 and 100 nm below the glass surface is likely to cause a destructive surface scratch eminently visible to the naked eye.

## Introduction

Scratch resistance of the surface of container glasses is one of the key challenges for the glass industry^1–3^. Container glass, e.g. baby food and milk bottles, is essentially soda-lime-silica (SLS) glass manufactured by the conventional melt-quenching process. The current industrial technology to enhance the scratch resistance is based on the deposition of a thin layer of tin oxide hot-end coating. A standard technique is to apply hot-end coating on the outer surface of container glass via chemical vapor deposition (CVD)^4^. The tin oxide coating acts as an anchoring agent and a primer for the cold-end coating, which is a thin layer of polymeric emulsion such as polyethylene and waxes. The flexible polymer chain primarily serves as a load dissipating (lubricating) agent during a scratching event to lower the coefficient of friction—consequently enhancing the scratch resistance of the glass surface^2,3,5,6^. This two-stage coating process is widely accepted by container glass-producing companies. Other coating systems, including SiO_2_-TiO_2_ and chromium thin films, were reported to favorably modify the SLS glass surface to enhance the abrasion resistance with low wear rates^7,8^.

However, the technical knowledge pertaining to the parental scientific background behind the formation of scratches on glass surfaces in terms of structural properties is still limited, with confined knowledge on the subject of the distribution of surface flaws on silicate glasses with relevance to brittle fracture and consequent degradation of mechanical strength^9–11^. Tempered glasses are known to be more sensitive to the visibility of scratches than their annealed counterparts^12^. The crack width of a typical scratch depends on the geometry of the indenter used, in addition to the applied normal load. The formation of radial, median and lateral crack systems induced by scratching with an indenter has been schematically explained. The effect of indenter geometry^13^ and applied normal load on the sequential regimes of plastic deformation (microductile), microcracking (radial cracks, median/lateral cracks), chipping and microabrasion phases (with increasing normal load) induced during and after unloading of indenter pertaining to scratch tests on SLS glass surfaces have gained attention in several publications^14–17^. Wondraczek and coworkers studied the dependence of scratch hardness on the composition of SLS glass and found increasing and decreasing trends of scratch hardness in the low load elastic–plastic deformation regime of 50–70 mN (absence of microscopic cracks) with increasing contents of CaO and Na_2_O, respectively^18^. It is equally noteworthy to consider the environment in which the scratch/wear tests are conducted, which has a major influence on the mechanism of cracking behavior governing the formation of a scratch on SLS glass surface, which is largely driven by stress corrosion cracking in humid atmosphere due to attack of water molecules on the (Si–O–Si) bridging oxygens to dissociate into silanol groups (SiOH) with consequent reduction of network connectivity^19–21^.

As far as the surface structural integrity of industrial container glasses is concerned, the SiO_2_-rich skin is known to play a critical role; the thickness of which, depends on the cooling rate during the melt-quenching process of product formation^22^. Mechanical injury to the glass skin is a cause of concern with respect to available openings in the form of microchannels for the migration of network modifier cations such as sodium, possible ion-exchange processes with atmospheric interactions and other detrimental features to dissolute and perturb the glass network.

The underlying principle behind the formation of an eminent scratch on glass surface visible to naked eyes is driven by the initiation and propagation of a crack induced by plastic deformation in association with chipping and micro abrasion instigated by the virtue of shear stress acting on the surface—accounting for a certain surface waviness and roughness^23,24^. The length, width and depth of the formed crack govern its characteristic orientation with respect to a direct correlation with the role of the surface structural network to dissipate or confine a scratch, as discussed in the next section. The optical phenomena in relevance with reflection and scattering of the photons of visible electromagnetic waves (400 nm to 700 nm) interacting with the electrons of the object of interest (indented grooves and chipped off materials on glass surface) are resolved and perceived by the color-sensitive receptors of our eyes in the form of a scratch^25^.

To the best of our knowledge, there is no information available on the surface structural behavior of the silicate network in terms of an elaborate analysis of network connectivity, which is responsible for triggering the formation of a visible scratch. To discover different ways of enhancing scratch resistance on glass surfaces, it is essential to understand the underlying mechanism in terms of structural modifications governing the basic principle of scratch formation. In this paper, an attempt has been endeavored to illustrate the structurally “weakening elements” that are accountable for the formation of scratches on SLS glass surfaces. Experimental evidence by virtue of X-ray photoelectron spectroscopy (XPS) and Raman spectroscopy has been analyzed in addition to complementary qualitative optical micrographs to propose the fundamental science governing the formation of scratches on SLS glass surfaces.

## Results and discussion

The formation of a scratch is governed by the nature and live state of the SLS glass surface just before the event of scratching. The structural orientation of the silicate network plays a crucial role in addition to the distribution of network modifiers—governing the integrity of the surface required for absorption and dissipation of a dynamically applied load. If the externally applied force (sheer stress) is opposed by the glass surface instead of dissipating it to a larger area (wide scratch) in the neighboring vicinity of the position of the application of load, the appearance of a scratch is thought to be relatively more abrupt, dictated by the sheer dominance of the surface. A harder surface is thus likely to oppose the applied load more effectively (lower scratch depth) than a relatively softer counterpart to promote the instigation of higher stress concentration within a smaller confined volumetric zone. This is proposed to lead to better visibility and prominence of a formed scratch on a harder surface, which succeeds the context of the current discussion.

**Figure 1:**
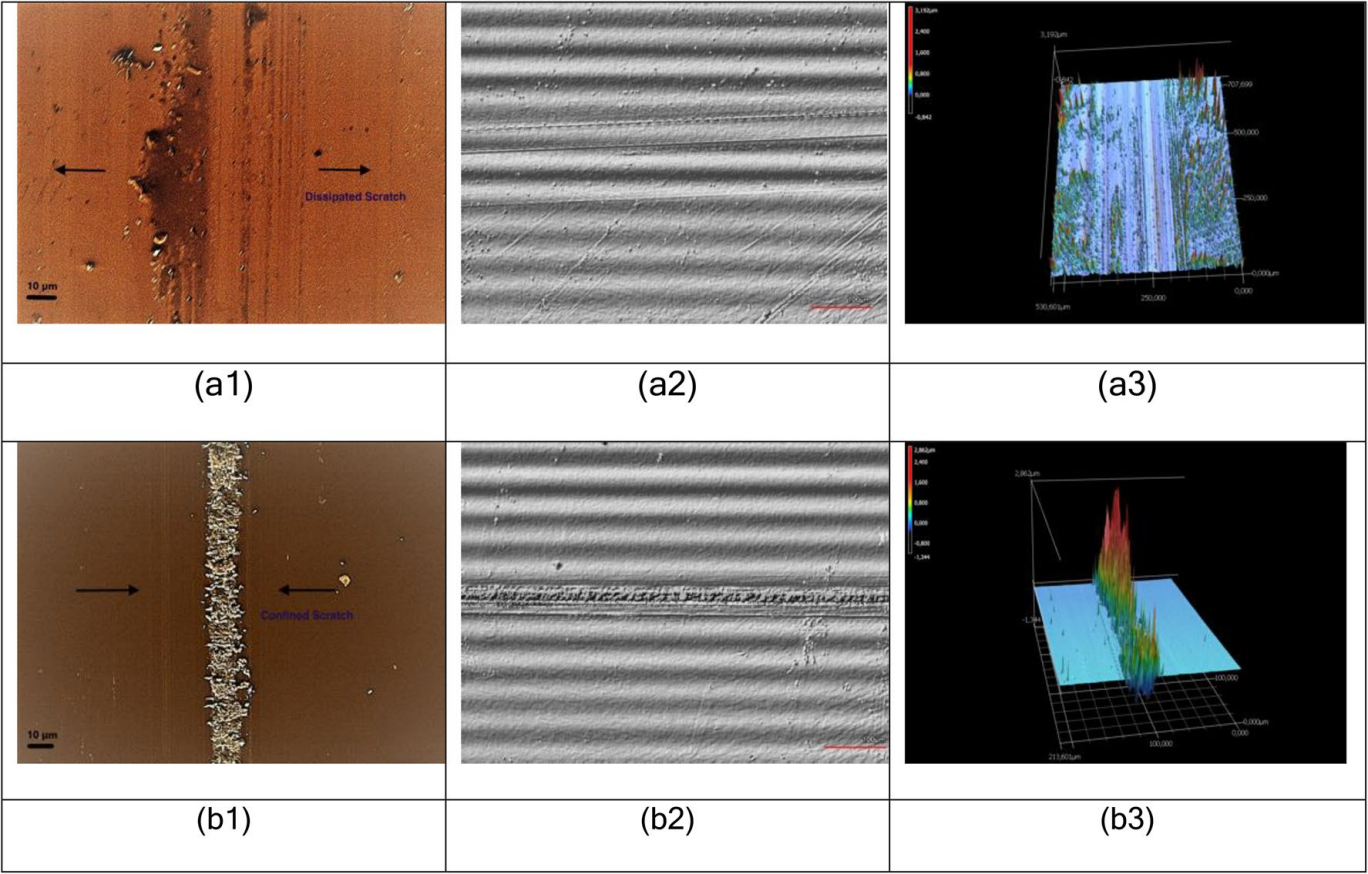
SEM, DIC and LSM (projected) images of scratches generated at 5 N on **(a)** untreated **(b)** heat-treated (510 °C, 30 min) SLS glass surfaces.

For the sake of a systematic study of the relationship of structural-mechanical property governing the formation of visible scratches on SLS glass surface, a fixed high normal load of 5 N was applied on the surface to generate scratches in the micro-abrasive regime^17^ at a fixed high scratching speed of 25 mm/s in a uniformly controlled manner, before and after sub-T_g_ (T_g_: glass transition temperature) heat-treatment for subsequent surface structural characterizations. The glass transition region of the studied SLS glass was measured to be confined between 559 °C and 575 °C by the change of slope in dilatometric study, at a heating rate of 5 °C/min. Figure 1a portrays the two-dimensional scanning electron microscopic (SEM), three-dimensional laser scanning microscopic (LSM) and differential interference contrast (DIC) images of a scratch generated on an untreated SLS surface. Figure 1b represents the SEM, LSM and DIC images of a scratch formed at the same load of 5 N on an SLS surface heat-treated close to T_g_ at 510 °C for 30 min. It is clearly visible that the scratch on the heat-treated surface is more concentrated within a confined region with a high volume of material pile-up (chipping), leading to higher prominence of its visibility in comparison to the untreated counterpart, which seems to be dissipated to a larger width with scattered debris. Increasing the normal load would increase the width and depth of the scratches with higher wear volumes^26^.

**Figure 2:**
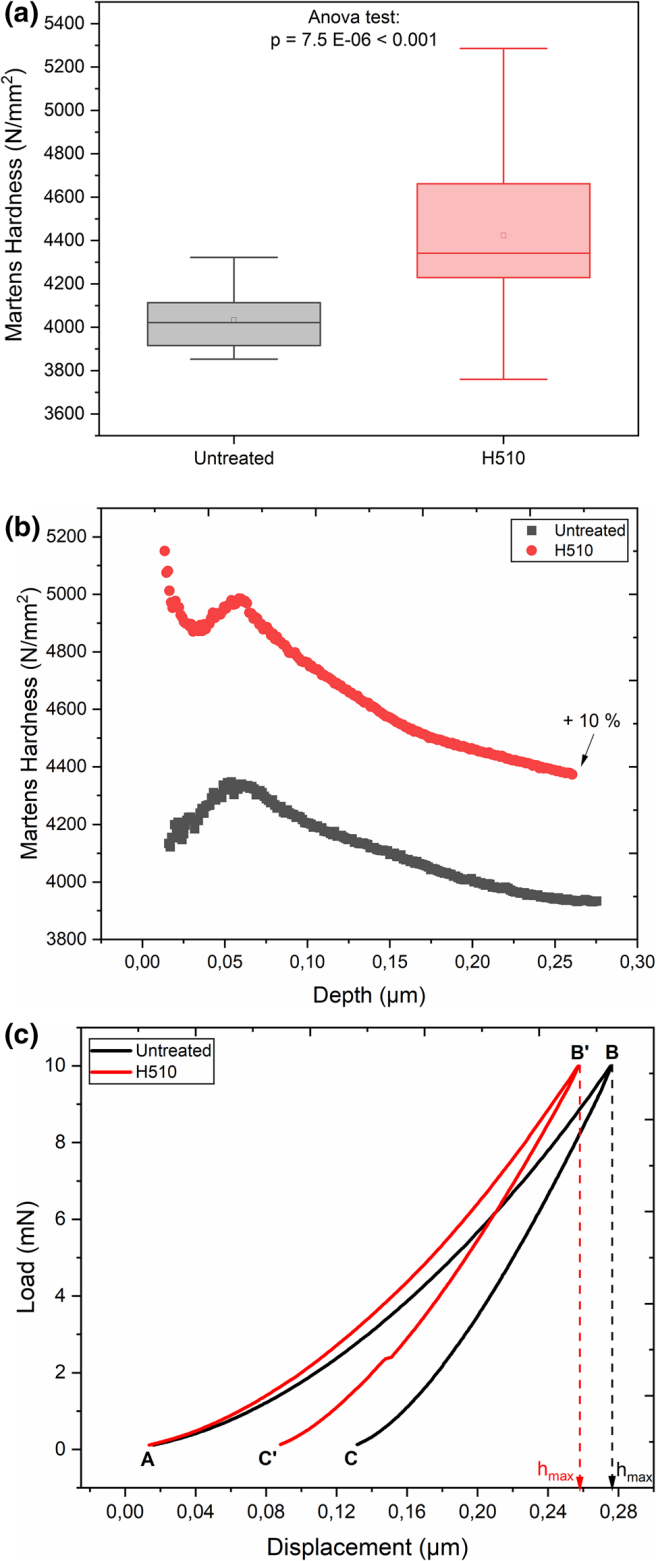
**(a)** Statistical analysis of hardness of untreated and heat-treated glass surfaces at a load of 10 mN for 16 indentations performed across an array of 4 × 4. The p-value reported by ANOVA test indicates that the means are significantly different at the 0.001 level. **(b)** Comparative illustration of the mean of variation in hardness as a function of depth from the untreated and heat-treated SLS glass surfaces. The indentation size effect (ISE) due to dislocation strengthening necessary to accommodate plastic deformation, in addition to friction between the indenter and specimen surface^39,40^, may not be neglected at shallow depths below 100 nm. The Martens hardness in the ordinate (y-axis) contains an axis-break. **(c)** Corresponding mean load–displacement curves illustrating the loading cycle (AB: untreated and AB’: H510) and unloading cycle representative of elastic recovery (BC: untreated and B’C’: H510). The serration observed in the unloading cycle (B’C’) of the H510 specimen reflects local relaxation processes during elastic recovery, which is predominantly more active during the unloading process than during loading^41^.

The increased vulnerability of the heat-treated surface to visible scratches might be related to a modified subsurface hardness. The comparative variation of hardness as a function of depth from the glass surfaces (mean of 16 indentations) observed by instrumented indentation is illustrated in Fig. 2b. The corresponding mean load–displacement curves shown in Fig. 2c illustrate the loading cycle and unloading cycle, which is representative for the behavior of the glass network during penetration of the Vickers indenter. The ANOVA test in Fig. 2a confirms that the variation in hardness between the untreated and heat-treated surfaces is significant. The lower depth of maximum penetration (h_max_) of the H510 surface at the same load of 10 mN relative to the untreated surface is accountable for its higher Martens hardness. It is essential to point out that we did not observe any distinguishable difference in hardness between the heat-treated and untreated specimens at loads greater than 10 mN—corresponding to higher depths of penetration over 300 nm (bulk).

The scientific reason in terms of structural modification for a 10% enhancement in the surface hardness of a sub-T_g_ heat-treated specimen are revealed by structural analysis via XPS studies in the top 10 nm of the glass surface, and by Raman spectroscopy in the bulk structure, which are presented in the following sections.

### Investigation of surface silicate structure by X-ray photoelectron spectroscopy (~ 0–100 nm)

**Figure 3:**
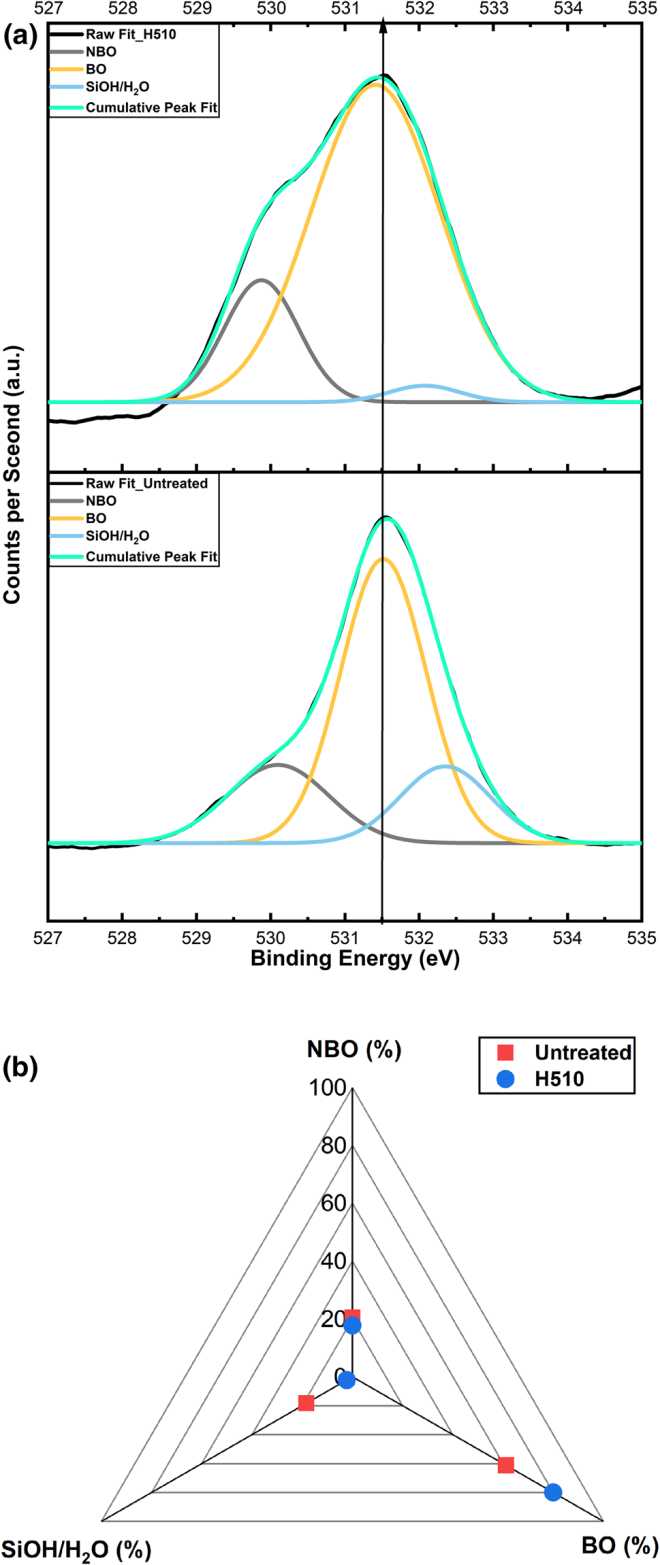
XPS results of untreated and heat-treated SLS surfaces (0–5 nm): **(a)** 100% Gaussian deconvolution of the O1s orbital and **(b)** comparative illustration of the distribution of bridging oxygen (BO), non-bridging oxygen (NBO) and SiOH/H_2_O species on both surfaces.

X-ray photoelectron spectroscopy (XPS) is a very powerful surface-sensitive technique to determine localized atomic bonding environments in addition to elemental depth profiling analysis in nanometer ranges of depth below the glass surface. Initially, an XPS measurement was performed on the top surface without any sputtering. The information obtained is confined within a depth of 5 nm corresponding to this measurement. Figure 3 portrays the XPS results of the surfaces of untreated and heat-treated specimens (before the scratch test) in terms of O1s peak deconvolution, centered around their corresponding binding energies (assignment of spectral peak fits:^27,28^). The O1s peak fittings were performed freely without any constraints in accordance with Nesbitt et al.^28^ to report the least squares best fits. The corresponding binding energies, full width at half maxima (FWHM) and normalized integrated peak areas of the deconvoluted peaks are tabulated in Table 1. The comparative differences in the concentrations of non-bridging oxygens (NBOs) and bridging oxygens (BOs) substantiate critical evidence of the orientation of the silicate structure (network connectivity) in addition to the distribution of network modifiers, which may govern the mechanistic driving force of the formation of a scratch on the SLS glass surface. Figure 3a shows a comparative deconvoluted O1s illustration with respect to a clear distinction between untreated and heat-treated surfaces in terms of a noteworthy difference in the concentration of BOs and NBOs corresponding to their respective binding energies, ensuing the subsequent illustration. The normalized integrated peak areas were used for quantitative computation of the concentration of BOs and NBOs present on the surfaces of both specimens. The evaluated concentrations are comparatively plotted in Fig. 3b.

**Table 1:** Binding energy, FWHM and % area of deconvoluted O1s peaks of untreated and heat-treated surfaces.

Specimen	NBO			BO			SiOH/H_2_O		
	BE (eV)	FWHM (eV)	Area (%)	BE (eV)	FWHM (eV)	Area (%)	BE (eV)	FWHM (eV)	Area (%)
H510	529.87	1.17	17.77	531.41	2.03	80	532.07	1.11	2.23
Untreated	530.09	1.61	20.35	531.51	1.35	62.09	532.35	1.42	17.56

The presence of a relatively high concentration of bridging oxygens on the surface (within a depth of 5 nm) of the heat-treated specimen is clearly noticeable. The scientific reason in terms of structural modification of the sub-T_g_ heat-treated specimen may be hypothesized to be the repolymerization of NBOs of vicinal silanols in a subsurface layer, referred to as “inner skin”. The presence of repolymerized Q^4^ units would result to a strengthened silicate network, to corroborate the evidence of higher (nano) hardness, as observed by instrumented indentation at a load of 10 mN. Moreover, the presence of relatively lower concentrations of “mechanically weakening elements”, namely, NBOs and SiOH/H_2_O species, on an SLS surface heat-treated near T_g_ further justifies the network strengthening effect induced by the virtue of thermal treatment.

Thus, a combination of a high concentration of bridging oxygens and a low population of mechanically weakening elements up to a depth of about 5 nm indicated a strong and rigid network possessed by the heat-treated surface; in other words, the surface porosity^29^ was hypothetically reduced by sub-T_g_ heat treatment. The presence of free volume within the silicate network associated with inherent voids may be perceived as surface porosity in terms of the availability of a micro channelized pathway^22^ for the propagation of a crack from the injured surface skin through the depth of the network. A strong and rigid network with higher concentration of repolymerized BOs formed by condensation of vicinal hydroxyls^27^ and associated with Q^4^ units in the heat-treated surface—ensures a lower availability of free volume within the surface network to contribute to higher hardness. In contrast, the higher concentration of NBOs associated with silanol groups at this depth of the untreated surface is accountable for its lower hardness, owing to a weaker structural network with respect to lower degree of polymerization^30^.

Subsequently, the structural network connectivity was further investigated as a function of depth from the glass surface by sequential sputtering of Ar^+^, to probe the depth of the near-surface region. Assuming an estimated etching rate of approximately 1 nm/min with Ar^+^ sputtering – considering the report of Yamanaka et al. (50 nm/hr.) with respect to XPS studies on SLS float glass surfaces^32^, it can be assumed that the information was obtained up to a depth of approximately 100 nm with 110 min of Ar^+^ sputtering at 5 kV, concerning the SLS glass used in this study. Although argon ion sputtering was reported to cause potential surface damage in terms of possible migration of mobile alkali ions in addition to possible surface modification by long duration XPS experiments^33,34^, it is still a widely used method for XPS depth profiling on glass surfaces and should not affect the analysis of the O1s orbital presented in this work, pertaining to an experimental Ar^+^ sputtering time of less than 2 h at 5 kV.

**Figure 4:**
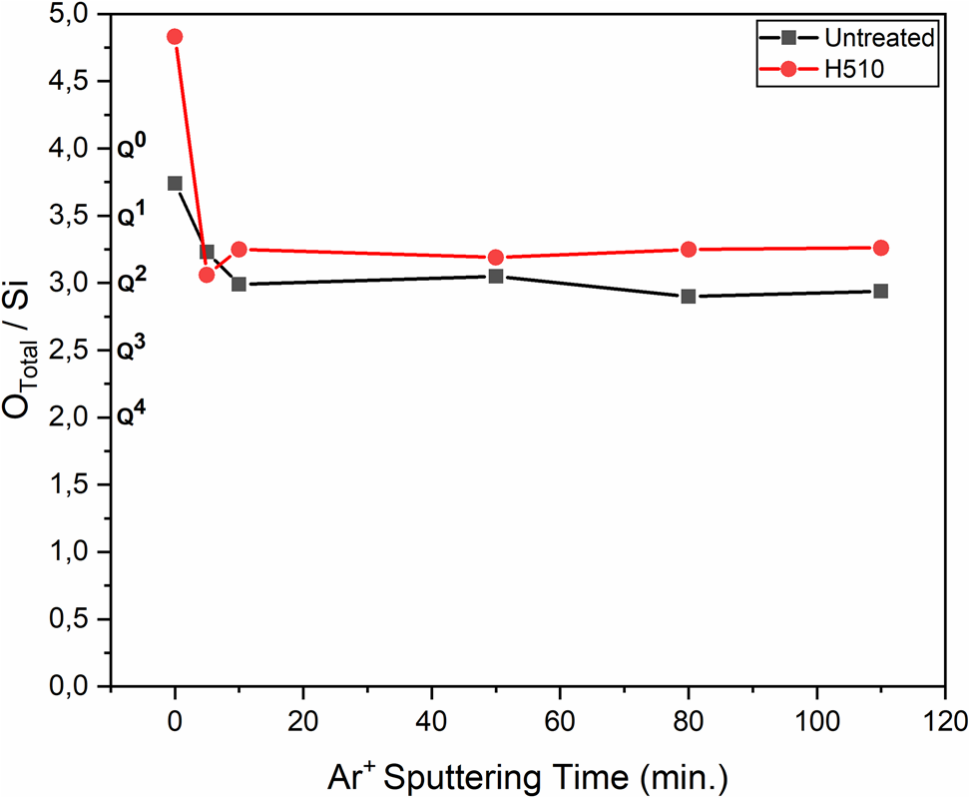
Comparative variation of the total oxygen (O_Total_) to silicon (Si) atomic ratio as a function of Ar^+^ sputtering time (depth range ~ 0–100 nm) for untreated and heat-treated specimens. The relative sensitivity factor (RSF) was taken into consideration. Connecting lines act as guideline to the eye.

The total concentration of the contribution of all oxygen speciations of O1s spectral line was calculated for each step of XPS measurement and put in relation to the concentration of Si2p, expressed as O_Total_/Si atomic ratio in Fig. 4. The O_Total_/Si ratio of the heat-treated specimen seemed to attain a saturation between 3 and 3.5—indicative of the predominant presence of Q^1^ and Q^2^ species. The untreated specimen, on the other hand, saturated at a ratio close to 3, indicating a major dominance by a potentially high population of Q^2^ species.

It should be noted that the data point corresponding to “0 min” indicates a surface measurement without sputtering, while the depth of information is confined within a depth of 5 nm below the surface. The H510 surface—corresponding to this first measurement—seemed to contain an abundance of free oxygen (O^2−^), as indicated by a very high O_Total_/Si ratio of 4.7. These “free” oxygens are considered to occupy the voids within the repolymerized Q^4^ units present on the heat-treated surface, in accordance with XPS results of heat-treated glass surfaces reported by Banerjee et al.^27^. We complement their findings by observing high concentrations of free oxygens on the surface by our experimental investigations. Moreover, Nesbitt et al. also reported the presence of free oxygens in the form of O^2−^ on the glass surface by complementary XPS and NMR studies^28^. Nonetheless, charge neutrality must be maintained, and is thought to be controlled by proximate modifier cations. The second probable reason to account for an O/Si ratio greater than 4, could be the presence of physiosorbed or chemisorbed water from the atmosphere before subjecting the heat-treated specimen to XPS analysis. It is noteworthy that the binding energy of the Na1s spectral line is close to 1075 eV, in contrast to a low binding energy of around 530 eV for the O1s spectral line, which theoretically implies that the photoelectrons of Na1s and O1s are ejected from slightly different depths (of the order of probably a couple of nanometers, which may still not be neglected) corresponding to any data point of sputtering. Hence, we preferred to avoid a comparative analysis of Na1s and O1s orbitals.

**Figure 5:**
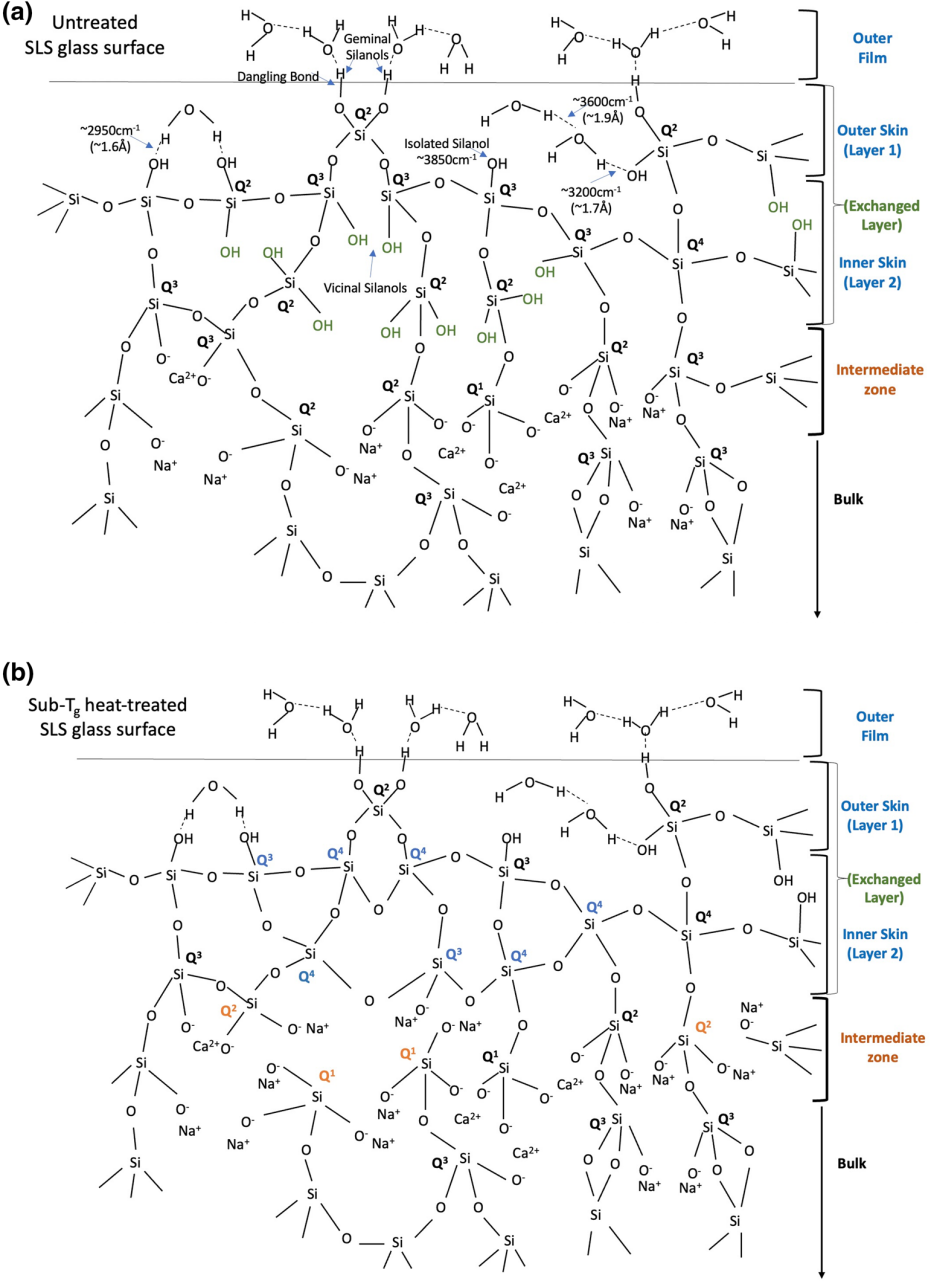
Typical surface structural schematic representations of SLS glass **(a)** untreated **(b)** heat-treated at 510 °C for 30 min. The surface OH groups pertaining to physisorbed and chemisorbed water molecules in the outer skin (layer 1) were assigned by ATR-IR signals. The inner skin (layer 2) containing vicinal silanol groups highlighted in green in (**a**), repolymerizes into stronger Q^4^ units—evidenced by higher concentration of bridging oxygens obtained by XPS O1s in Fig. 3—to contribute to subsurface hardening of heat-treated glass. The depth of the SiO_2_-rich glass skin (exchanged layer) is on the order of few nanometers, which depends on the thermal history and atmospheric storage history, among other surface-influential factors. The altered Q^n^ notations, specifically to Q^4^, in the exchanged layer after sub-T_g_ heat-treatment is highlighted in blue. The mechanically weakening structural characteristic of the intermediate zone with respect to higher O_Total_/Si ratio obtained in the heat-treated surface in Fig. 4 is critical to scratch formation. The network depolymerization after heat-treatment in this thin subsurface layer is marked by the transformation into weaker Q^1^ and Q^2^ units (highlighted in orange in (**b**)), accompanied by higher concentration of NBOs.

The aforementioned findings clearly demonstrate that the surface of the H510 specimen up to about 5 nm was mechanically stronger than the untreated surface with respect to higher concentration of bridging oxygens—reflected by a schematic representation in Fig. 5, indicated by the formation of repolymerized bridging oxygens formed by condensation of vicinal hydroxyls and associated with Q^4^ units in the heat-treated surface, here called “exchanged layer”.

The reverse holds true for the zone confined between the depth range of 5 nm and 100 nm, which is critical. The sub-T_g_ heat treated glass structure was found to be mechanically weaker in this region, herein called “intermediate zone”, owing to higher O_Total_/Si ratio. This depth is critical to propagation of cracks from a surface flaw during a scratching event because the size of the cavities in the vicinity of crack tips is reported to be in the same range on the order of nanometers^31^. Crack growth is known to occur by nucleation, growth and coalescence of cavities at the crack tips, leading to the propagation of cracks through the thin subsurface zone confined between 5 and 100 nm, which is evidenced to be critically defined by a relatively weaker network consisting of Q^1^ and Q^2^ species than the bulk of the network that is known to be populated by stronger Q^3^ units (Raman stretch ≈ 1090 cm^−1^).

It is noteworthy to mention that although the atomic ratio of O_Total_/Si corresponding to the data point of ‘0 min’ (surface measurement without sputtering) for the H510 surface is considerably high, it does not indicate a structurally weakened network due to compensation by a high population of BOs, as described earlier.

### Investigation of bulk silicate structure by Raman spectroscopy (~ 1–5 µm)

**Figure 6:**
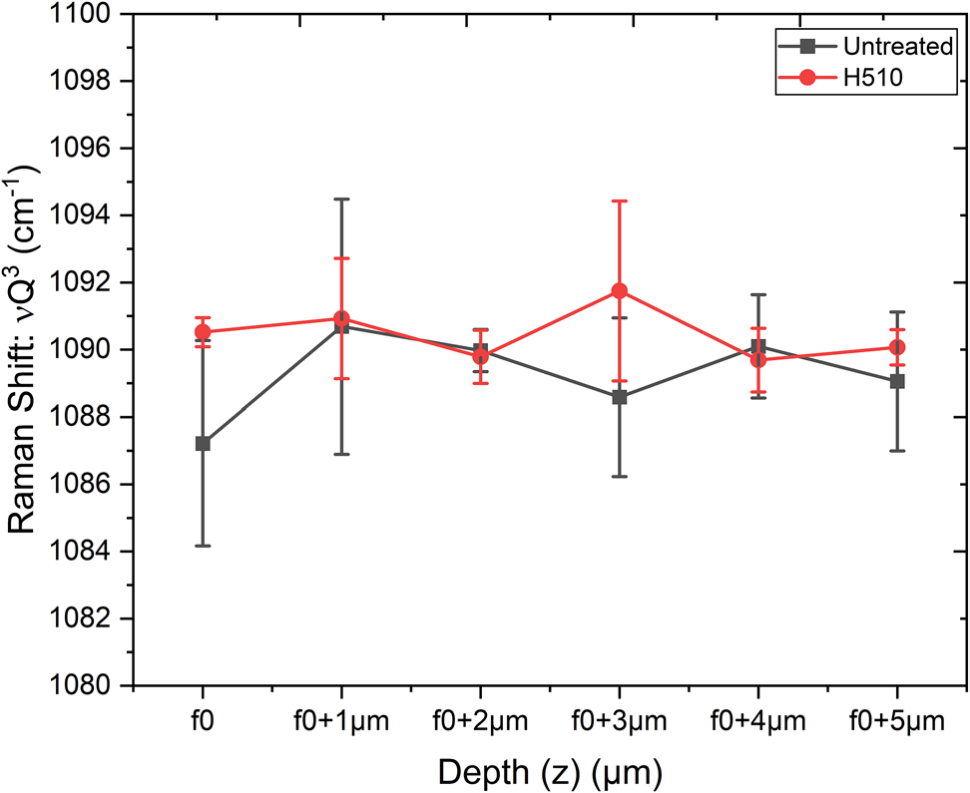
Comparative Raman shifts of the position of the high-frequency Q^3^ stretching band with depths up to 5 µm pertaining to untreated and heat-treated specimens. The respective standard deviations were derived from five measurements of Raman shifts as a function of depth, for each category. Depth Resolution ~ 1 µm.

Having studied the interesting silicate structural changes in the depth range of 5 to 100 nm below the glass surface by XPS, it was essential to investigate the silicate structure in micrometer ranges of depth (before the scratch test), which can be conveniently probed by confocal Raman spectroscopy with a high-resolution z-scan through the depth. A depth resolution of approximately 1 µm was obtained with careful optimization of different optical parameters while bearing in mind the index of refraction. The theoretical spatial (x–y) resolution was 500 nm with a laser spot diameter of 1 µm on the specimen surface. The high frequency (HF) broad band centered around 1090 cm^−1^ is commonly known to be attributed to the stretching vibrations of Q^3^ species^35–37^. The noteworthy finding of this study is the distribution of the Raman shift of the Q^3^ band with respect to the scattering (shift) of its position with depth from the glass surface, micrometer by micrometer. The untreated specimen showed a scattered distribution of Q^3^ shift including the standard deviations considering five measurements at each point in depth, compared to the heat-treated specimen when scanned up to 5 µm from the point of focus on the top surface f_0_ (penetration depth of 532-nm laser into silicon is reported to be 0.7 µm^38^). This is illustrated in Fig. 6. The Raman shift of the HF Q^3^ band was proposed to be a function of variation in the Si–O-Si bond angle and Si–O bond length in different studies concerned with ion-exchanged SLS glasses^36,37^. However, the scattered distribution of the shift observed in this work is thought to qualitatively indicate a more pronounced variation of the Si–O–Si bond angle with depth for the untreated specimen, probably indicating a relatively more stabilized silicate network (with respect to lower variation of bond angle) for the heat-treated specimen in the bulk (µm range), due to its limited Q^3^ shift with depth.

**Figure 7:**
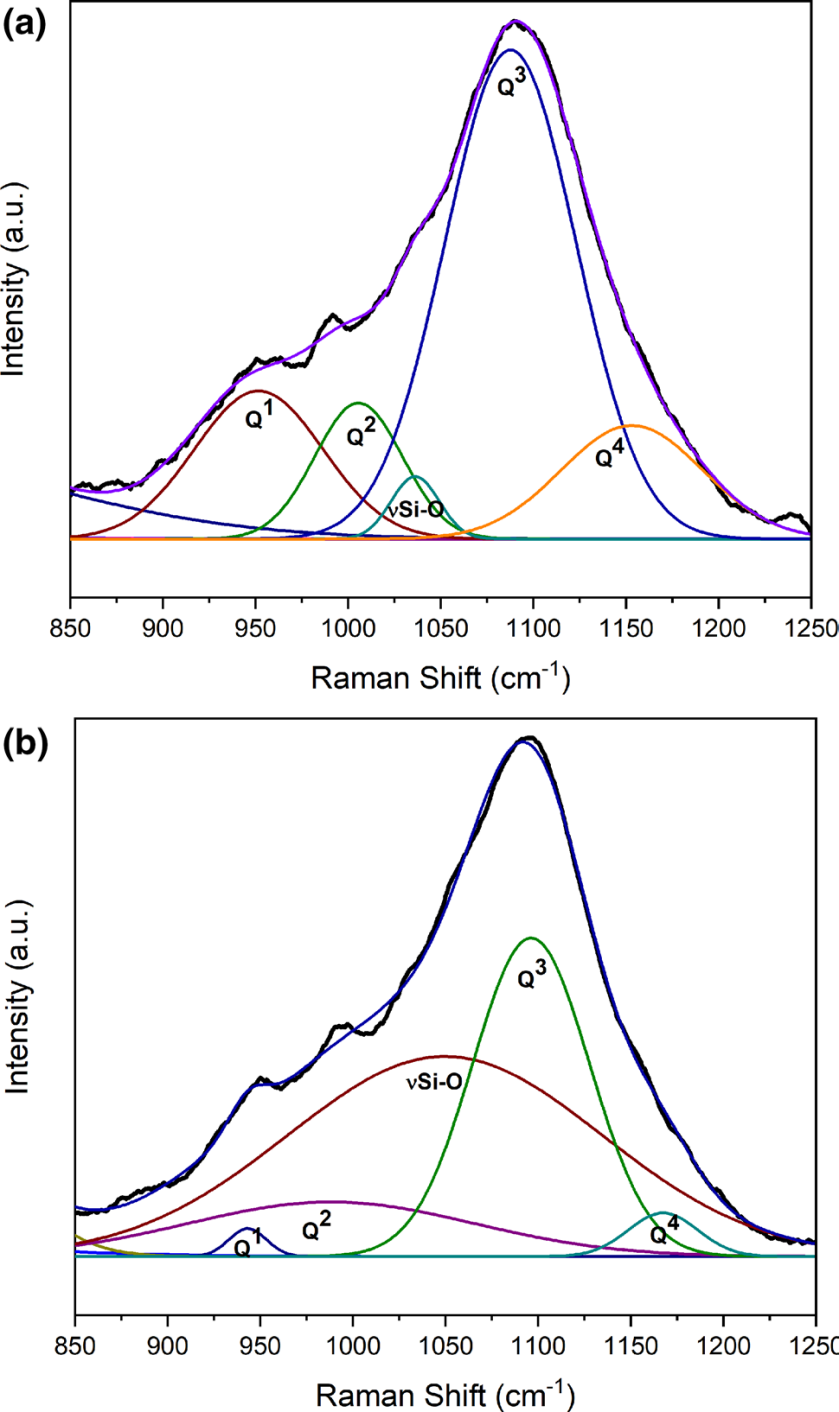
Gaussian deconvolution of the HF Raman band (850 cm^−1^ to 1250 cm ^−1^) indicating the overlapping peaks corresponding to Q^n^ species present in the scratched network of **(a)** untreated and **(b)** heat-treated surfaces (R-square > 0.99 in both spectral fits).

The Raman spectra of the HF stretching band (850 cm^−1^ to 1250 cm^−1^), taken directly on the scratched grooves of both specimen surfaces, are shown in Fig. 7. Gaussian deconvolution was performed after necessary processing of the spectra to report the best generated fit of the overlapping peaks (R-square > 0.99), which were assigned to the stretching vibrations of Q^1^, Q^2^, Si–O, Q^3^ and Q^4^ species—corresponding to approximately 950 cm^−1^, 990 cm^−1^, 1040 cm^−1^, 1090 cm^−1^ and 1150 cm^−1^, respectively^36^. The surface scratch network on the untreated specimen seemed to contain more Q^1^ units (mechanically weakening entity), defined by a larger area under the shoulder peak at approximately 950 cm^−1^ (16.7%), relative to the H510 counterpart (only 1%), where the full width at half maxima (FWHM) was almost four times. This corroborates the preceding observation of a lower variation in bond angle with depth from the heat-treated surface before scratching (stabilized initial network). The lower randomization of the initial silicate network as a function of depth in the bulk of the H510 structure evidenced before scratch (Fig. 6) is proposed to be accountable for lower concentration of weakened structural elements of Q^1^ in the scratched groove observed as a significant difference in the peak-fitted spectra in Fig. 7b. The cause of lower concentration of stronger Q^4^ species in the scratched groove of heat-treated structure may be correlated to a relatively destructed network in the scratched groove, accompanied by high volume of material pile-up observed on the heat-treated surface in Fig. 1b. The third shoulder corresponding to approximately 1040 cm^−1^ was assigned to the stretching vibration of a depolymerized Si–O unit, which was slightly debatable to be assigned to any specific Q^n^ species^35,36^. A comparative illustration of the normalized integrated areas under the individual peaks (expressed in %) and the corresponding FWHM values is tabulated in Table 2. However, the drawback associated with Raman spectroscopy is its inability for an accurate quantitative analysis, although the area under the peaks can be compared within a particular spectrum to draw apparent interpretive conclusions.

**Table 2:** The % Area and FWHM of overlapping peaks present in deconvoluted HF Raman band of respective surface scratch grooves.

Peak label	Normalized integrated area (%)		FWHM (cm^−1^)	
	Untreated	Heat-treated	Untreated	Heat-treated
Q^1^	16.7	1	82	24
Q^2^	10.4	13	56	182
ν Si–O	2.6	52.5	31	200
Q^3^	56.2	31	83	84
Q^4^	14.1	2.5	90	44

## Summary of XPS and Raman investigations

The XPS investigations were focused on unveiling the surface structural network connectivity up to a depth of about 100 nm. This thin subsurface layer was found to be characteristic of a mechanically-weaker network, predominantly consisting of Q^1^ and Q^2^ species—dictated by the O_total_/Si atomic ratio hovering around 3. The silicate structure of the bulk network investigated by Raman spectroscopy predominantly consisted of stronger Q^3^ units–dictated by the pronounced intensity of the high frequency stretching band at around 1090 cm^−1^.

Considering the comparative evidence off XPS and Raman investigations of the untreated and sub-T_g_ heat-treated SLS glass, we propose the following difference in gradient of strength (with respect to silicate network connectivity concerning the distribution of BOs, NBOs, possible variation of Si–O-Si bond angles and Q^n^ species with depth) of the silicate network as a function of depth from the glass surface:

- *Untreated* weak (up to 5 nm)—strong (5 to 100 nm)—weak (1 to 5 µm);
- *Sub-T*_*g*_* heat-treated* strong (up to 5 nm)—weak (5 to 100 nm)—strong (1 to 5 µm).

The stronger surface structural network (up to 5 nm) evidenced by XPS and the lower randomness of the bulk network (1 µm to 5 µm) indicated by Raman—contribute to higher subsurface hardness of the sub-T_g_ heat-treated glass (H510). However, the existence of a mechanically-weaker ‘intermediate zone’ between 5 and 100 nm with respect to higher O_total_/Si ratio (lower degree of polymerization) in the H510 specimen is proposed to be the detrimental cause of visible scratch formation. Thus, a sub-T_g_ heat-treated SLS glass is more vulnerable and sensitive to visibility of scratches than the untreated counterpart. This is apparently homologous to a previous report which discovered tempered glasses to be more sensitive to scratches than the annealed counterpart^12^.

To generalize the root cause of scratch formation on SLS surfaces, the network connectivity of the thin subsurface layer between 5 and 100 nm is proposed to be the critical zone of consideration. A weaker structural network in this region dominated by Q^1^ species–is expected to contribute to easy propagation of a surface-initiated crack to the depth of the stronger bulk network that is known to be abundantly populated by stronger Q^3^ species. Any surface injuries will be subsequently accompanied by higher scratch depths, wear volume and material pile up.

### Further work

The future study is oriented towards extending the XPS measurements for several hours accompanied by Ar^+^ etching with low energy gas cluster ion beam (GCIB) to potentially ensure that the glass network is not affected in spite of prolonged exposure. This may give rise to the possibility to probe further into the depth of the glass network to complementarily correlate the distribution of Q^n^ species obtained by Raman investigations in the bulk structure of the solid specimens. In addition, the investigations of the effect of atomistic surface roughness and waviness on scratch formation is considered essential to subsequently propose a surface structural model of SLS glass, which could possibly further explain the parent cause of scratches induced during the handling and storage of container glass bottles in reality.

## Conclusion

Determination of the root cause of scratch formation on the soda-lime-silica glass surface was correlated to the network connectivity of the silicate structure. For the sake of a systematic scientific investigation, a simple linear scratch test was performed on an untreated and a sub-T_g_ heat-treated (510 °C, 30 min) SLS glass surface by a controlled, fixed load of 5 N. Qualitative investigation of the scratches was performed by virtue of scanning electron microstructures and laser scanning micrographs. It was observed that the heat-treated surface was more sensitive to visible formation of scratches than the untreated counterpart. Nanoindentation was performed before the scratch test to investigate the (static) surface hardness at a load of 10 mN; the results indicated a 10% enhancement in surface hardness restricted to a depth of about 300 nm below the surface of the heat-treated specimen.

An elaborate investigation of the silicate structure was performed before scratch testing to determine the structural cause of the formation of scratches. X-ray photoelectron spectroscopic results showed a higher concentration of bridging oxygens and a lower concentration of non-bridging oxygens on the heat-treated surface in comparison to the untreated counterpart (up to a depth of about 5 nm), which is indicative of a strengthened network to contribute to higher subsurface hardness. Regarding silicate network connectivity, the untreated specimen showed Q^2^ saturation (O_Total_/Si ≈ 3) up to a depth of approximately 100 nm; while the heat-treated counterpart saturated at a slightly higher atomic ratio of around 3.3, indicating a high population of Q^1^ and Q^2^ species—characteristic of a mechanically-weakening near-surface network to increase the vulnerability of visible scratches. Confocal Raman spectroscopy (before the scratch test) showed a relatively lower scattering of the Q^3^ stretching band position as a function of depth from the heat-treated glass surface (up to approximately 5 µm), indicating a lower variation in the Si–O–Si bond angle and/or Si–O bond length in the region between 1 µm and 5 µm below the glass surface. Raman spectra taken over the scratched surfaces showed a relatively higher population of Q^1^ species associated with the scratched network on untreated specimen corresponding to the area under the shoulder peak around 950 cm^−1^, indicating a stronger silicate network of the scratched heat-treated surface, complimentarily attributed to higher surface hardness.

Overall, a silicate network possessing the following gradient of strength is proposed to be more vulnerable to visible surface scratches in terms of the distribution of Q^n^ species and SiOH/H_2_O species, among other accountable factors: strong (0–5 nm)—weak (5 nm to 100 nm)—strong (1 µm to 5 µm); the intermediate subsurface layer comprising of mechanically-weakening Q^1^ and Q^2^ species between 5 and 100 nm is the most critical zone.

## Methods

Soda-lime-silica container glass bottles with flat surfaces supplied by ‘Wiegand-Glas’ were used in this study. The surfaces of the bottles were without any hot-end and/or cold-end coatings. Cylindrical samples with a diameter of 25 mm and thickness of 3 mm were mechanically drilled out of the bottles by means of a wet-cutting process utilizing a diamond shaft. It was ensured that the surfaces of the obtained specimens (except the edges) were not mechanically injured during the drilling process. The samples were rinsed in distilled water, dipped in a static acetone bath for 15 min and again rinsed in distilled water before gently blow-drying the surface with nitrogen gas (producer: ‘Riessner Gase’; purity: 99.999%, humidity ≤ 5%) at room temperature. Some specimens that were characterized after the aforementioned process were labeled “untreated” specimens. A few samples were heat-treated at 510 °C for 30 min in a tube furnace (Carbolite HST 12/400), followed by naturally cooling to room temperature (labeled “H510”/“heat-treated”). The subsequent characterizations were performed within a day to avoid the effect of surface degradation (aging) by the attack of atmospheric moisture with long-term exposure, owing to the susceptibility of the outer skin of the glass surface (top 10 nm) to constant interaction with any surrounding environment.

The elemental composition of the bulk (depth over 1 µm) of untreated SLS glass consisted of (in atomic %) 50.7% Si, 22.0% O, 15.9% Ca, 8.9% Na, 1.4% Al and 1.1% K (EDX at 20 kV). The glass transition region (viscosity≈ 10^11^ Pa.s to 10^12.3^ Pa.s) of the untreated SLS specimen was measured to be confined within the range of 559 °C to 575 °C by dilatometric analysis (Netzsch 402 E/7/E-Py) at a heating rate of 5 °C/min.

### Characterization techniques

A field emission scanning electron microscope (Model: Zeiss Sigma 300 VP) was used with secondary electron signals at a low accelerating voltage of 3 kV to generate surface-sensitive microstructures.

A laser scanning microscope (Model: Keyence VK-X1100) equipped with a class 2 laser of wavelength 404 nm (output power: 1 mW) was used to obtain three-dimensional projected images and differential interference contrast (DIC) images of the scratches on the glass surface by a non-contact mode of measurement.

X-ray photoelectron spectroscopy (XPS) was performed by a PHI Versa Probe III spectrometer with an Al K alpha source (1486.6 eV). The target current on the specimen holder was 3 µA, while the focus beam current at the Faraday cup was 302 nA. Surface charge neutralization was performed by virtue of a dual beam charge neutralization system that utilizes both a cold cathode detector hood source and a very low energy ion source (< 10 eV) to provide turnkey charge neutralization. The pass energy was 26 eV, and the spectral resolution was approximately 0.2 eV. The samples (before scratch tests) were introduced to an ultrahigh vacuum (UHV) XPS chamber at a pressure on the order of 10^–9^ mbar to investigate the structural network connectivity of both the untreated and heat-treated specimens up to a depth of approximately 100 nm for the sake of scientific interpretation of the root cause of scratch formation in terms of the difference in surface structural orientation in both samples.

Initially, surface measurements were performed on each of the untreated and heat-treated SLS specimens to determine the concentrations of bridging oxygens (BOs), non-bridging oxygens (NBOs) and SiOH/H_2_O species by (100% Gaussian) deconvolution of the O1s peak. The depth from which the photoelectrons are ejected for this measurement is confined within a region of 1–3 nm below the glass surface. The surface XPS measurement was subsequently followed by sequential XPS measurements at specific time intervals accompanied by Ar^+^ sputtering at 5 kV (to remove layer by layer from the glass surface in five steps) to determine the O_total_/Si atomic ratio (Q^n^ network connectivity in relevance with obtained coordination number; where ‘n’ indicates the number of BOs linked to a silica tertrahedron) as a function of depth from the glass surface for both the untreated and heat-treated specimens. Elemental composition profiles and corresponding binding energy curves were processed and extracted with MultiPak software after Shirley background correction for subsequent Gaussian spectral fits.

Raman spectroscopy was carried out by a Bruker Senterra II using a 532-nm laser source. A 50 × objective with a numerical aperture of 0.65 was used to obtain a laser beam spot diameter close to 1 µm on the specimen surface. The groove density of the diffraction grating was 1200 lines/mm. The laser power was set to 12.5 mW with an aperture of 25 µm and an integration time of 150 s to obtain an optimum signal-to-noise ratio with a spectral resolution of 1.5 cm^−1^_._ A z-scan was performed as a function of depth from the glass surface with an approximate depth resolution of 1 µm by stepwise moving the stage away from the focus point on the specimen surface (f_0_) while bearing in mind the index of refraction.

Instrumented indentation was performed on an SLS glass surface by a Fischerscope HM2000 at a load of 10 mN with a load application time of 20 s. The loading rate was fixed to “d(Sqrt)F/dt = constant”. Sixteen indentations were performed across an array of 4 × 4 on each specimen. The corresponding hardness vs depth curves were dynamically recorded by “WIN-HCU” application software by virtue of continuous generation of load–displacement curves throughout the depth of penetration. Martens hardness (HM) was expressed in N/mm^2^ (HM = F/k*h_max_^2^, where k = 26.43 for the Vickers indenter with a face angle of 136°, F is the applied load and h_max_ is the maximum depth of penetration).

Scratch tests on the SLS glass surface were performed by an Erichsen Lineartester Model 249 using a tip 16/505 according to ISO 1518-1, with scratch stylus ‘B’ having a hemispherical hard-metal tip of diameter 1 mm. A fixed (high) load of 5 N was applied during the dynamic scratch tests through the length (diameter) of the glass sample at a high speed of 25 mm/s to form straight, fine surface scratches in the microabrasion regime, visible to the naked eye. The device enabled constant load application in a uniformly controlled manner during the scratch tests. The experiments were performed in a laboratory with a surrounding temperature of approximately 25 °C and relative humidity in the range of approximately 60% to 65%.
